# Pre-operative gluteus medius tendon degeneration and its impact on strength and functional ability one year after total hip replacement

**DOI:** 10.1080/07853890.2024.2388701

**Published:** 2024-08-14

**Authors:** Johanna Juhlin, Ninni Sernert, Kristina Åhlund

**Affiliations:** a Department of Physiotherapy, NU Hospital Group, Trollhättan/Uddevalla, Sweden; b Institute of Clinical Science, Department of Orthopaedics, Sahlgrenska Academy, University of Gothenburg, Gothenburg, Sweden; c Department of Research and Development, NU Hospital Group, Trollhättan/Uddevalla, Sweden; d Department of Health Sciences, University West, Trollhättan, Sweden

**Keywords:** Functional ability, gluteus medius, hip osteoarthritis, muscle strength, rehabilitation, tendon degeneration, total hip replacement

## Abstract

**Objective:**

Hip osteoarthritis is a common cause of disability and surgery is often unavoidable. Patient satisfaction is high and functional ability improves after surgery. However, residual impairment and pain are common. Degenerative changes in tendons and muscles are probable causes. The aim of this study is to investigate gluteus medius (GMED) tendon degeneration in relation to muscle strength, physical function and walking distance before and one year after total hip replacement.

**Material and methods:**

In total, 18 patients were examined pre- and post-operatively, of whom 15 were available in the final analysis. Muscle strength, physical function and walking distance were assessed. Tendon biopsies were assessed microscopically, and the total degeneration score (TDS) was calculated.

**Results:**

A correlation between the TDS and muscle strength was found for the hamstrings, GMED and quadriceps pre- or post-operatively. No correlations were found between the TDS and functional ability. Functional ability and muscle strength improved significantly after surgery.

**Conclusion:**

Our results indicate a correlation between tendon degeneration and the muscle strength of the hip and knee in patients with hip OA and one year after THR. To minimise post-operative residual discomfort, rehabilitation programs should probably be modified over time to match the pre- and post-operative needs. Further studies are needed.

This study was registered at https://www.researchweb.org/is/vgr/project/279039 (in Swedish).

## KEY MESSAGES

- There are negative correlations, which suggest patterns between degeneration in the GMED tendon and muscle strength in the muscles acting around the hip in patients with hip OA, before and after THR.
- The strength training of muscles acting around the hip joint may need to be adjusted before and after THR.

## Introduction

Osteoarthritis (OA) is the most common chronic joint disease in Sweden [1] and in the rest of the world [2] and the most common cause of disability in the elderly, with large societal costs as a result [1]. The most affected joints are the knee, hand and hip [2, 3]. Basic treatment, according to Swedish National Guidelines, comprises physical training, patient education, medication and, if needed, weight loss. When these are no longer sufficient as treatment, total hip replacement (THR) is the remaining option [1]. However, lingering pain and residual perceived functional impairment are common one year or more after knee or hip replacement surgery [4]. Swedish national guidelines recommend in-hospital early mobilisation, functional training and adapted exercise immediately after surgery. Patients are then recommended to continue their rehabilitation in a primary care physiotherapy setting within 4 weeks from hospital discharge [5].

The gluteus medius muscle (GMED) is our primary abductor and the most important hip stabiliser during the stance phase of the gait cycle [6]. It is, together with sufficient strength in the quadriceps muscle, hamstrings muscle and other muscles acting around the hip, important during functional activities such as rising, standing and walking [7, 8]. It has previously been established that patients with hip OA have a reduced cross-sectional area in the hip and thigh muscles, and reduced muscle strength around the hip and knee, including GMED [9–12], poorer functional capacity and are less physically active compared with healthy adults [10]. Degenerative changes in several of the surrounding tendons and muscles, including GMED, of the affected joint are suggested as one cause of reduced muscle strength both pre- and post-operatively [11,13,14] and a probable cause of residual post-operative discomfort [14]. Degenerative changes, from tendinosis to complete GMED tendon rupture, have been demonstrated in patients with hip OA both pre- and post-operatively [14–17], as well as in healthy elderly individuals, with the progression of tendon degeneration with increasing age [18]. Previous research has shown that the quality of the GMED tendon is significant for the occurrence of pain and lameness one year after THR in symptomatic patients [14]. Furthermore, Rosinsky et al. showed that patients with tendon degeneration verified with MRI, but no pre-operative functional signs of pathology of the abductor tendons (GMED and gluteus minimus), showed worse pain and patient satisfaction than those without tendon pathology 2 years post-operatively [19]. This indicates the importance of identifying these patients at an early stage. Hendry et al. point out that the actual occurrence of the rupture of structures involved in hip abduction may be underdiagnosed [20], but it has also previously been shown that muscle strength training has positive effects on tendon properties in the elderly [21]. To our knowledge, it is not known yet, to what degree degeneration in the GMED tendon affects other major muscles around the hip and its relation to strength, physical function and walking in patients having hip replacement surgery. It is therefore of interest to analyse the relationship between these variables. The hypothesis is that degenerative changes in the GMED tendon is related to muscle strength in GMED, hamstrings and quadriceps and physical function including walking. As a result, the aim of this study is to analyse pre-operative degenerative changes in the GMED tendon in relation to muscle strength, physical function and walking distance, before and one year after elective THR.

## Materials and methods

This is a clinical longitudinal study, derived from a large research project by Ibrahim et al. which was conducted at a medium-sized hospital in western Sweden between 2016 and 2019 [22].

The study was registered at https://www.researchweb.org/is/vgr/project/279039 (in Swedish). The patients received information about the study orally and in writing and informed consent was obtained. In accordance with the Declaration of Helsinki, all participants were protected from injury and retained their right to privacy and to informed consent. All data were treated confidentially [23]. The study was approved by the Regional Ethics Review Board in Gothenburg, date 4 March 2013, diary no: 381-15, with an additional application for this specific sub-study, diary no: T359-17.

In the present study, all the patients with primary hip OA, planned for surgery, were consecutively asked to participate. The exclusion criteria were: previous hip surgery, disease or other conditions affecting the neuromuscular function of the lower extremities, osteonecrosis of the femoral head, frail patients with multimorbidity or severely ill patients, dementia or other cognitive impairment, widespread malignancy and systemic corticosteroid treatment for more than three months.

Pre-operatively all patients received written and verbal information from a physiotherapist about the post-operative rehabilitation and hip precautions including no hip flexion beyond 90°, no internal rotation and no adduction for 3 months.

All patients received in-hospital post-operative rehabilitation according to the national guidelines including training to become independent in activities of daily living (ADL) and exercises for strength and mobility. At discharge, 2 days after surgery, the patients were instructed to initiate rehabilitation in a primary care unit, of their choice, within 4–6 weeks.

## Measurements

Muscle strength, mobility, walking distance and hip range of motion (ROM) were assessed at the hospital pre-operatively and one year post-operatively. All measurements were performed by the same experienced physiotherapist, who was not involved in the patient’s rehabilitation process.

For the TDS-scoring two tissue samples (0.5 × 0.5 cm), from the GMED tendon were harvested from each patient, at the tendon’s insertion site on trochanter major, during surgery. They were then prepared analysed using a light microscope as thoroughly described elsewhere [22]. The histological evaluations were performed by a pathologist and an orthopaedic surgeon. Fibre structure, cellularity and vascularity and the presence of glycosaminoglycans (GAGs) were classified according to a four-point (ranging 0–3), semi-quantitative scoring system. The total degeneration score (TDS) was calculated by adding the mean values of the two biopsies for the four elements, resulting in values ranging from 0 (no degeneration) to 12 (extremely large degeneration) [22,24]. This scoring system has demonstrated satisfactory intra-observer reliability [24].

Isometric muscle strength was measured in hip abduction, knee extension and knee flexion, using a hand-held dynamometer (HHD); MicrofetII (Hoggan Scientific LLC, Salt Lake City, Utah, USA). The HHD has excellent intra-rater (ICC 0.89–0.99) [25–27] and inter-rater reliability (ICC 0.76–0.97) [25,27,28] and validity [29]. Starting positions were chosen based on recommendations from the manufacturer [30, 31]. Hip abduction was measured with the patient supine and the dynamometer placed about 5 cm above the femoral condyle [31]. Knee extension and knee flexion were measured with the patient sitting on an examination table without back support and with the legs hanging freely. A fixation strap was placed over the thighs. The HHD was placed on the tibia, when testing the quadriceps, and on the calf, when testing the hamstrings, at a height of approximately two fingers above the malleolus [30]. For patients with extensive quadriceps strength, an extra fixation strap was placed around the examination table and the patient’s distal tibia. The MicroFetII was then placed between the tibia and the fixation strap. The patient was asked to push as hard as possible for four seconds and then relax. Each muscle group was tested three times and the patient was allowed 30 s of rest between each try. The highest value was recorded. To minimise the risk of using arm and trunk strength, the arms were kept crossed over the chest. The leg that was not planned for surgery was tested first.

Timed Up and Go (TUG) was chosen to quantify functional mobility. It has been proven to be a reliable and valid instrument, suitable for assessing mobility in orthopaedic patients [32], and it can easily be included in clinical practice [32,33]. TUG shows excellent reliability (ICC 0.96) for THA patients, where improvements over time of 1.62 s or more are considered clinically relevant [34]. The patients performed the test twice. The time for the best attempt was recorded.

To quantify walking distance, the 6 min Walk Test (6MWT) was performed. It is highly reliable in assessing walking capacity in patients after THR (ICC 0.96) and has been shown to detect even small changes post-operatively, where an improvement of just over 10 metres is considered clinically relevant [35]. Instruction and performance followed the American Thoracic Society guidelines for the test [36].

Statistical analyses were performed using the IBM Statistical Package for the Social Sciences 25 (SPSS) licensed for the NU Hospital Group. Demographic data are reported with the mean (SD), median (min-max) and percentage (%). As the study population is small, non-parametric analyses were chosen. Change over time was calculated with Wilcoxon’s Signed Ranks Test. Correlation analyses were performed with Spearman’s rho and interpreted as shown in Table 1 [37]. Significance level 0.05.

**Table 1.** Correlation interpretation.

Correlation coefficient	Interpretation
0.00–0.10	Negligible correlation
0.10–0.39	Weak correlation
0.40–0.69	Moderate correlation
0.70–0.89	Strong correlation
0.89–1.00	Very strong correlation

Our study is based on data from the study by Ibrahim et al. [22], and therefore there was no possibility to influence the sample size and power in relation to the present measurements. Our study sample may therefore be considered a sample of convenience as it is somewhat underpowered.

## Results

**Figure 1.**
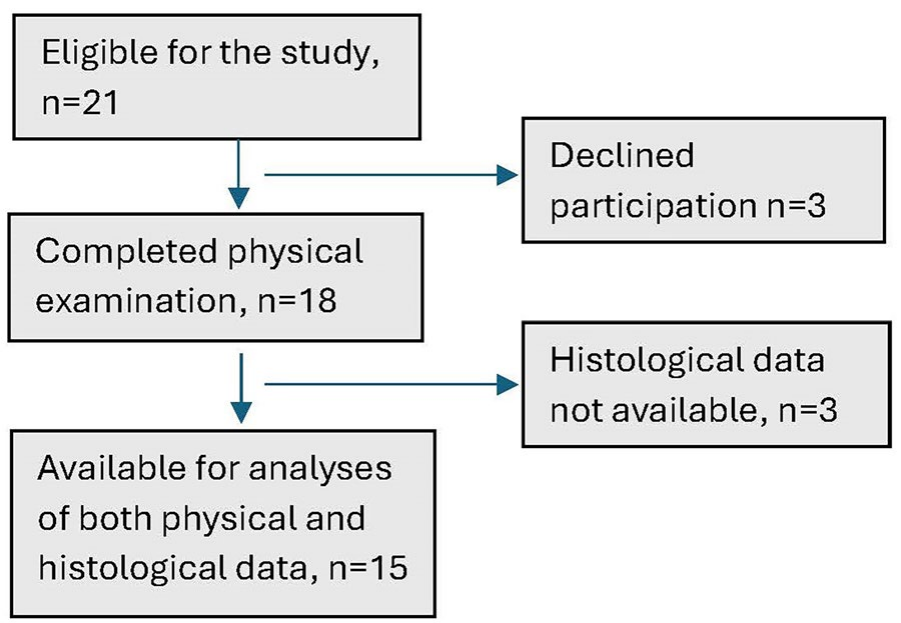
Flow chart.

In total, 18 patients were included in the analysis, see flowchart (Figure 1). Demographic data are reported in Table 2.

**Table 2.** Descriptive statistics pre-operatively.

	*n*	
*Age*	18	
Median (min-max)		69.5 (51–81)
Mean (SD)		67.5 (8.5)
*BMI* (kg/m²)	15	
Median (min–max)		28 (24–34)
Mean (SD)		28.5 (3.6)
*Sex*	18	
Women *n* (%)		8 (44)
Men *n* (%)		10 (56)
*Walking aid*	18	
Yes *n* (%)		6 (33)
No *n* (%)		12 (67)
*Arthrosis educational course*	18	
Yes *n* (%)		10 (56)
No *n* (%)		8 (44)

BMI: Body Mass Index.

Arthrosis educational course: a theoretical and practical education held by physiotherapists and occupational therapists in primary care units and part of the basic treatment available to everyone diagnosed with arthrosis.

**Figure 2.**
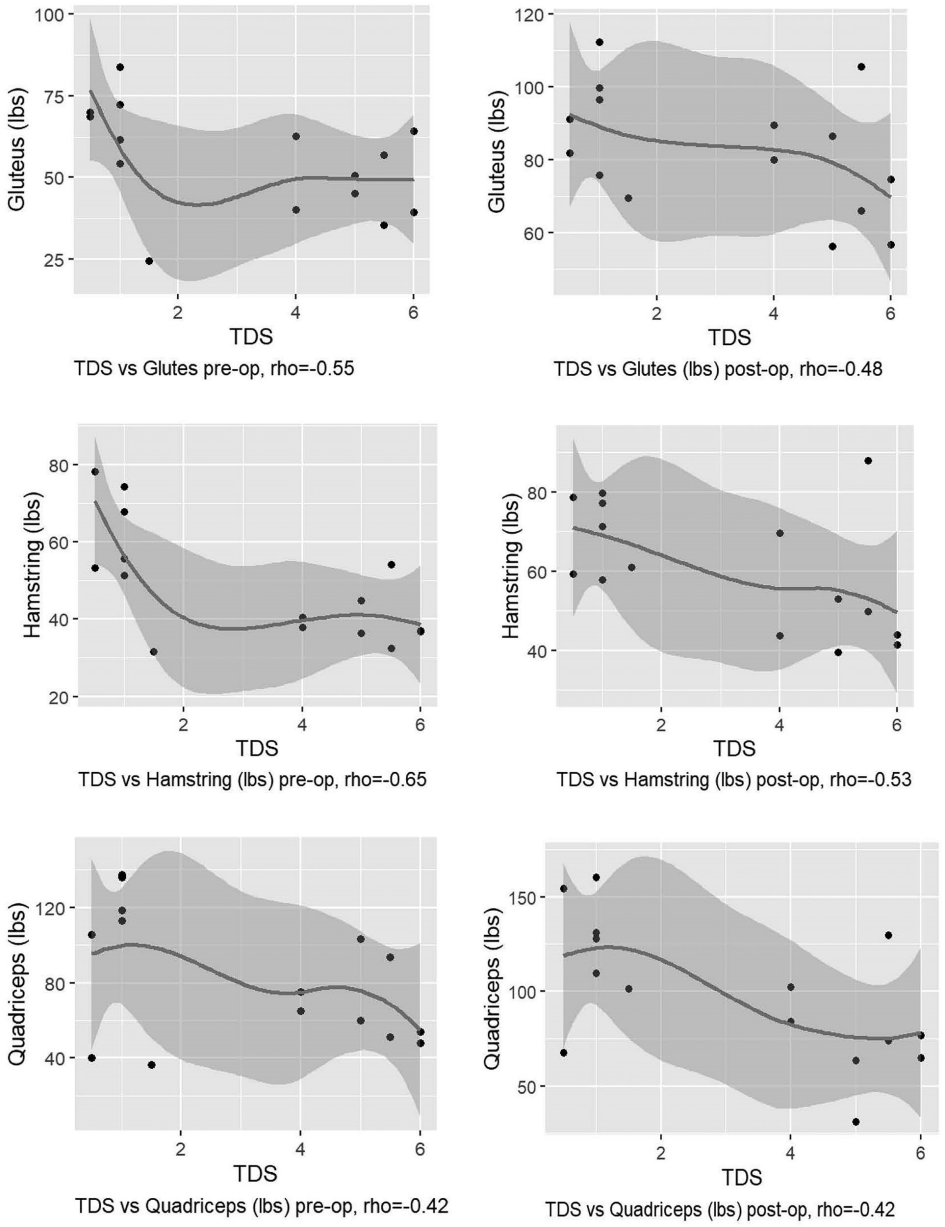
Scatterplots of pre-operative TDS-values vs. muscle strength in GMED, hamstrings and the quadriceps muscles, pre-and post-operatively. In the scatterplots the line is fitted with a smoother (the R function ‘geom_smooth’) which fits the best line to the data using a local polynomial surface. Note that Spearman method of correlation is a measure of monotonicity and not a linear relation. The shaded region is the confidence interval for the fitted line. Abbreviations: Gluteus – Glutes Medius muscle, hamstring – the hamstring muscles, quadriceps – quadriceps muscles, TDS – total degeneration score, pre-op – preoperative values, post-op – postoperative values, rho­ spearman’s correlation coefficient, vs – versus, lbs – pounds

The correlation between the TDS in the GMED tendon and muscle strength, mobility (TUG) and walking distance (6MWT) both pre- and post-operatively is reported in Table 3. A moderate negative correlation between the TDS and muscle strength was seen in the hamstrings, in the operated leg, both pre- and post-operatively (Spearman’s rho = −0.65 and Spearman’s rho = −0.53 respectively). For the GMED, a moderate negative correlation with the TDS was seen pre-operatively (Spearman’s rho = −0.55). For the quadriceps, a moderate negative correlation was seen post-operatively (Spearman’s rho = −0.53). To illustrate the results of the correlations a scatterplot of TDS vs GMED (Ibs), hamstring (lbs) and the quadriceps (Ibs) are included, please see Figure 2.

**Table 3.** Correlation between GMED tendon degeneration and muscle strength, physical ability and walking distance.

	*n* = 15			Total Degeneration Score (TDS)
Quadriceps pre-op			Spearman’s rho *P*-value	0.42 0.12
Quadriceps post-op			Spearman’s rho *P*-value	−0.53 0.04*
Hamstrings pre-op			Spearman’s rho *P*-value	−0.65 0.01*
Hamstrings post-op			Spearman’s rho *P*-value	−0.53 0.04*
Gluteus medius pre-op			Spearman’s rho *P*-value	−0.55 0.04*
Gluteus medius post-op			Spearman’s rho *P*-value	0.48 0.07
Timed Up and Go (TUG) pre-op			Spearman’s rho *P*-value	0.24 0.39
Timed Up and Go (TUG) post-op			Spearman’s rho *P*-value	0.21 0.46
6 min Walk Test (6MWT) pre-op			Spearman’s rho *P*-value	−0.06 0.84
6 min Walk Test (6MWT) post-op			Spearman’s rho *P*-value	−0.07 0.79

Correlations with a *p*-value <0.05 are marked with *

Negligible or weak correlations were seen between the degree of degeneration in the GMED tendon (TDS) and mobility (TUG) or walking distance (6MWT) both pre- and post-operatively. However, pre-operatively moderate correlations were shown between GMED strength and functional tests (TUG: Spearman’s rho = −0.49, 6MWT: Spearman’s rho = 0.45), which were not seen post-operatively.

Strength in the hamstrings (*p* < 0.01) and GMED (*p* < 0.01), but not the quadriceps, improved over time. In addition, functional ability (TUG) and walking distance (6MWT) improved over time after surgery compared with pre-operative values (*p* < 0.01, *p* < 0.01 respectively). This is reported in Table 4.

**Table 4.** Change in strength, physical ability and walking distance before and one year after surgery.

	*n*	Median (min–max)	Mean (SD)	*P*-value
*Quadriceps*	18			
Strength pre-op (lb)		62.4 (23.4–137.2)	76.2 (35.29)	
Strength post-op (lb)		80.4 (31.2–160.4)	90.53 (38.27)	
*P*-value				0.25
*Hamstrings*	18			
Strength pre-op (lb)		39.1 (16.5–78.1)	44.98 (16.45)	
Strength post-op (lb)		55.45 (34.9–88.0)	57.3 (16.63)	
*P*-value				<0.01*
*Gluteus medius*	18			
Strength pre-op (lb)		54.15 (24.6–83.8)	53.1 (16.1)	
Strength post-op (lb)		77.95 (28.6–112.4)	76.2 (22.5)	
*P*-value				<0.01*
*Timed Up and Go (TUG)*	18			
Pre-op (sec)		9.16 (4.35–21.34)	9.86 (4.40)	
Post-op (sec)		7.17 (4.60–12.93)	7.76 (2.11)	
*P*-value				0.01*
*6 Minute Walk Test (6MWT)*	18			
Pre-op (m)		410 (120–657)	419 (131)	
Post-op (m)		516 (309–766)	503 (119)	
*P*-value				<0.01*

lb: pounds.

sec: seconds.

m: metres.

*P=/<0.05.

## Discussion

The results in our study show a moderate correlation between degeneration in the GMED tendon and pre-operative GMED muscle strength. Furthermore, correlations were found between degeneration in the GMED tendon and muscles interacting with GMED in functional activities and walking, i.e. hamstrings pre- and post-operatively and quadriceps post-operatively. The present study also shows that muscle strength improved one year post-operatively compared with pre-operative values in the affected leg.

From a rehabilitation perspective, this knowledge can provide information on how degeneration in the GMED tendon affect the strength in major hip and thigh muscles and how they recover after THR. Ibrahim, et al. noted that patients who underwent a revision surgery of the hip had a more pronounced degeneration in the GMED tendon, compared to patients who underwent primary THR, which indicates that the tendon did not recover after a first hip replacement [22]. This suggests that the tendon may not easily recover after THR and rehabilitative focus should probably be placed on improving muscular function, without overloading the tendon.

It appears that GMED muscle strength improves most which, in this case, may be explained by poor pre-operative strength and/or pain, and pre-operative degeneration of the muscle and tendon. However, there were moderate correlations between muscle strength and functional tests. It is consistent with a previous study that presents similar pre-operative results regarding the relationship between strength and functional tests [38]. Furthermore, no correlations were seen between degeneration in the GMED tendon and the functional measurements used in the study. However, physical activities and walking are complex functions not only involving muscle strength but also requiring postural stability, co-ordination and balance involving other body functions.

Information about how strength in hip and thigh muscles change after THR and persist over time may guide physiotherapists in the planning of pre- and post-operative rehabilitation programs. In addition to the rehabilitation suggested by Swedish National Guidelines [5], our results emphasize the importance of GMED strength training in the pre-operative rehabilitation program, quadriceps strength post-operatively and hamstrings strength training throughout the rehabilitation period. This might hopefully provide the opportunity to affect both functional ability and pain.

This study has several strengths. To our knowledge, this is the first study analysing the association between pre-operative microscopic degeneration of the GMED tendon and muscle strength and functional outcome measurements in patients with hip OA and after THR. Secondly, the post-operative follow-up took place over a long period of time. It is already well known that large improvements occur during the early post-operative phase [39–41], but the effect of prosthetic surgery and following rehabilitation over time is not as well explored [42]. Another strength is the fact that the same senior physiotherapist conducted all the tests both pre- and post-operatively, contributing to high reliability in general and in strength measurements in particular. An HHD was used in this study, as it is easy to learn how to use it at a relatively low cost, compared with other devices measuring muscle strength [29], and it can therefore easily be used in a clinical setting. HHDs are regarded as both intra- and inter-rater reliable by some [25, 26], while others recommend the same examiner for repeat tests [28], which was the case in our study. There are, however, also limitations to this study. Firstly, since the study is underpowered one should be careful when interpreting the size differences between the significant correlation coefficients. However, several of the results are quite strong and they do suggest a pattern between strength and TDS. Secondly, pain was not accounted for in this study. Another limitation is the lack of a control group or comparison to the unaffected leg in the strength measurements, which could have been used as reference values. At follow-up, all patients were asked whether they participated in postoperative rehabilitation in primary care, but not what type of rehabilitation and to what extent. It would probably have been valuable if variables describing each patient’s post-operative rehabilitation were included in the study. The lack of this may be seen as a shortcoming, as the current results cannot be discussed from that perspective.

To capture a holistic perspective on the rehabilitation of patients who undergo THR, future and larger studies are needed. They may focus on providing further information about the degeneration of tendons in relation to physical function, muscle strength, lameness, different patient reported outcome measurements (PROMS) and detailed description of rehabilitation protocols in the primary care setting.

In conclusion: the results of this study indicate that there were moderate negative correlations between the degeneration of the GMED tendon and muscle strength of the major muscles acting around the hip, in patients who underwent THR, which persists one year after surgery. To minimize residual postoperative discomfort, rehabilitation programs may need to be modified over time to match pre- and postoperative needs.

## Data Availability

The data that support the findings of this study are available from the corresponding author, upon reasonable request.
